# Context-Sensitivity and Individual Differences in the Derivation of Scalar Implicature

**DOI:** 10.3389/fpsyg.2018.01720

**Published:** 2018-09-20

**Authors:** Xiao Yang, Utako Minai, Robert Fiorentino

**Affiliations:** ^1^Neurolinguistics and Language Processing Laboratory, Department of Linguistics, University of Kansas, Lawrence, KS, United States; ^2^Developmental Psycholinguistics Laboratory, Department of Linguistics, University of Kansas, Lawrence, KS, United States

**Keywords:** scalar implicature, question under discussion (QUD), individual differences, working memory, attentional control, Autism-Spectrum Quotient (AQ), pragmatic abilities

## Abstract

The derivation of scalar implicatures for the quantifier *some* has been widely studied to investigate the computation of pragmatically enriched meanings. For example, the sentence “I found some books” carries the semantic interpretation that at least one book was found, but its interpretation is often enriched to include the implicature that not all the books were found. The implicature is argued to be more likely to arise when it is relevant for addressing a question under discussion (QUD) in the context, e.g., when “I found some books” is uttered in response to “Did you find all the books?” as opposed to “Did you find any books?”. However, most experimental studies have not examined the influence of context on *some*, instead testing *some* sentences in isolation. Moreover, no study to our knowledge has examined individual differences in the ability to utilize context in interpreting *some*, whereas individual variation in deriving implicatures for *some* sentences in isolation is widely attested, with alternative proposals attributing this variation to individual differences in cognitive resources (e.g., working memory) or personality-based pragmatic abilities (e.g., as assessed by the Autism-Spectrum Quotient). The current study examined how context influences the interpretation of *some* in a story-sentence matching task, where participants rated *some* statements (“I cut some steaks”) uttered by one character, in response to another character’s question (QUD) that established the implicature as relevant (“Did you cut all the steaks?”) or irrelevant (“Did you cut any steaks?”). We also examined to what extent individuals’ sensitivity to QUD is modulated by individual differences via a battery of measures assessing cognitive resources, personality-based pragmatic abilities, and language abilities (which have been argued to modulate comprehension in other domains). Our results demonstrate that QUD affects the interpretation of *some*, and reveal that individual differences in sensitivity to QUD are modulated by both cognitive resources and personality-based pragmatic abilities. While previous studies have argued alternatively for cognitive resources or personality-based pragmatic abilities as important for deriving implicatures for *some* in isolation, we argue that arriving at a context-sensitive interpretation for *some* depends on both cognitive and personality-based properties of the individual.

## Introduction

In conversational exchanges, interlocutors commonly convey meanings which go beyond the literal semantic content of the utterance and require the generation of pragmatic inferences on the part of the comprehender. A widely researched phenomenon which is argued to involve pragmatic inferencing is the interpretation of the quantifier *some*. For instance, the utterance in (1) semantically entails that *at least one, and possibly all* of the students is hardworking, yet pragmatically the interpretation is often enriched with the implicature that *not all* of the students are hardworking (Noveck and Sperber, 2007; Katsos and Cummins, 2010).

(1) Some of the students are hardworking.
  Semantic entailment: At least one, and possibly all of the students is hardworking.
  Pragmatic implicature: Not all the students are hardworking.

The two readings differ in whether *all* is negated, since the semantic reading does not exclude the possibility that *all* may hold. In addition, the pragmatic implicature differs from the inherent semantic meaning, in that *not all* is cancellable but the semantic entailment *at least one* is not (Grice, 1989; see also Geurts, 2010), as shown in example (2) below:

(2) Non-cancellable semantic entailment:
  Some of the students are hardworking. #In fact, none of them are.
  Cancellable pragmatic implicature:
  Some of the students are hardworking. In fact, all of them are.

It has been argued that *some* is on a scale of quantifiers varying in informativity (i.e., how specific a quantifier is), ranging from the least to the most informative, e.g., *<some, many, all>* (Horn, 1972). Scalar implicature thus refers to the common intuition that a less informative item implies the negation of a more informative item on the scale, with *some* taken to imply *not all*. This meaning is often argued to arise due to interlocutors’ expectation that utterances shall be optimally informative, as formalized by Gricean maxims (Grice, 1989); thus, the comprehender can infer that the speaker must mean that the more informative term *all* does not apply if they opted to use the less informative term *some*.

It is important to point out, however, that *some* is not interpreted with the *not all* implicature in all cases. For example, linguistic analyses of *some* highlight that the likelihood of interpreting *some* with the implicature is heavily influenced by the broader context in which the *some* sentence appears (Roberts, 2004; see also Chierchia et al., 2012). One specific context-level factor that has been argued to play an important role in determining whether *some* is interpreted with the implicature is the question under discussion (QUD). QUD refers to the crucial issue in the discourse that is expected to be addressed by a relevant answer. The extent to which the *not all* implicature is realized in the interpretation of *some* is argued to depend on whether it is relevant under the current QUD (Roberts, 2004, 2012). Consider the following conversational exchanges, where the *some* utterances made by Speaker B in (3) and (4) are in response to different questions asked by Speaker A (examples adapted from Levinson, 2000; Politzer-Ahles and Fiorentino, 2013):

(3) Upper-bound QUD
  Speaker A: “Are all the students in this lab hardworking?”
  Speaker B: “Some of them are.”
(4) Lower-bound QUD
  Speaker A: “Is there any evidence against them?”
  Speaker B: “Some of their documents are forgeries.”

The reading that *not all* the students are hardworking strongly arises in B’s reply in (3). However, B’s reply in (4) can be felicitously interpreted without the *not all* implicature, as *at least one and possibly all* of their documents was a forgery. This is due to the difference in QUD in the two conversations, established by A’s questions. The QUD in (3) involves *all* the students, thus *some* in the reply should address A’s question and consequently be interpreted with the *not all* implicature. Conversely, the QUD in (4) involves whether there is *at least one* piece of evidence; thus *some* can be simply interpreted as *at least one* without the *not all* implicature, as *all* is irrelevant under A’s question. A QUD that highlights *all* and thus encourages the *not all* implicature is often termed as *upper-bound* (as in 3), while a QUD that does not encourage the implicature is termed as *lower-bound* (as in 4).

As is illustrated in examples (3) and (4) above, the QUD is often established in discourse through the linguistic utterances of the interlocutors in a conversation. These utterances may establish an issue that needs to be addressed, and thus indicate what is expected from an appropriate answer in the current discourse. In examples (3) and (4) above, for example, Speaker A establishes a QUD through a linguistic utterance whose properties (e.g., the choice of “all” versus “any”) set the stage for interpreting subsequent utterances containing *some* with or without the implicature. Thus, even though an answer sentence with *some* may be ambiguous by itself, contextual cues such as QUD are often provided by interlocutors which can be used to disambiguate the optimal reading of *some* in the discourse.

We would like to note that there are a range of theories that aim to account for how the *not all* implicature is generated, ranging from those positing the implicature is only generated when relevant (e.g., Relevance Theory approaches; Sperber and Wilson, 2002), to those in which the implicature is always generated, but may be canceled when not relevant (e.g., the Default view; Levinson, 2000), as well as views in which the *not all* interpretation is due to the presence/absence of a silent exhaustive focus operator (e.g., the Grammatical view; Chierchia, 2004; Chierchia et al., 2012) rather than to pragmatic inferencing. While these approaches differ with respect to the specific mechanism by which the *not all* meaning comes into consideration as part of the interpretation of *some*, they share the common assumption that context is important in determining whether *some* is ultimately interpreted with this meaning^1^. However, despite the crucial role of context highlighted in linguistic analyses of scalar implicature, only a handful of experimental studies have examined the extent to which comprehenders are indeed sensitive to context in interpreting *some*, with the majority of the literature instead testing *some* sentences in isolation, as we discuss below. Thus, the primary aim of the current study is to determine experimentally the extent to which comprehenders are sensitive to contextual information such as QUD and to characterize and account for the variability that individuals may show in sensitivity to context in interpreting *some*.

## Experimental Studies on Scalar Implicature

Studies on scalar implicature that do not manipulate context often test *some* sentences that are underinformative. These sentences are semantically true but pragmatically infelicitous based on, for example, world knowledge, such as the sentence in (5a) (examples from Noveck and Posada, 2003).

(5a) Underinformative:           Some dogs have paws.
(5b) True and informative:      Some people have pets.

In (5a), if *some* is interpreted as *some but not all*, the sentence is pragmatically infelicitous, as it would be more informative to use *all* instead (since all dogs have paws), although the sentence is semantically true (at least one dog has paws). Underinformative sentences can thus be used to test whether or not a scalar implicature has been generated: if the *not all* implicature is realized, the infelicity should lead to increased rates of rejection as compared to true and informative sentences such as (5b), in judgment tasks, and to evidence of processing disruption in online studies. Using this paradigm, studies have found that adult native speakers, when analyzed as a group, generally show sensitivity to pragmatic infelicity (e.g., Noveck and Posada, 2003; Bott and Noveck, 2004; De Neys and Schaeken, 2007; Huang and Snedeker, 2009; Hunt et al., 2013; Tomlinson et al., 2013).

However, one major drawback in studies establishing underinformativity based on world knowledge (as in example 5a) is that they require that participants draw on their world knowledge and verify if counterexamples exist to evaluate the sentences (e.g., dogs that have no paws). Judgments may thus depend on participants’ ability to consult the relevant world knowledge, and may vary based on participants’ beliefs about how typical the world under discussion is when they are presented with these odd utterances (see Degen et al., 2015). Another drawback is that effects of underinformativity can be confounded with lexical differences across conditions. Notice that (5a) for example contains the lexical items *dogs* and *paws*, while (5b) contains *people* and *pets* (see, e.g., Nieuwland et al., 2010 for discussion regarding this concern). Issues regarding world knowledge do not arise in variants of the underinformativity approach that provide the information needed to determine the felicity of the *some* sentences. This is often done by presenting a visual display with a number of objects and then asking participants to evaluate a *some* sentence about these objects (e.g., Huang and Snedeker, 2009; Hunt et al., 2013; Antoniou et al., 2016; among others). However, a general limitation of studies testing *some* sentences presented in isolation is that they do not directly target the comprehension of *some* within a broader context that provides information indicating whether the implicature is relevant, which might better approximate how comprehenders typically must interpret *some* sentences during everyday language use.

Another finding from this line of research on *some* in isolation is that individual native speakers have been shown to vary greatly from one another in terms of whether the *not all* implicature is derived. Many studies have revealed that native speakers generally fall into two groups: one group of speakers that consistently rejects underinformative sentences, suggesting that they interpret *some* pragmatically, and another group that consistently accepts underinformative sentences, suggesting that they interpret *some* semantically, without realizing the implicature (Noveck and Posada, 2003; Bott and Noveck, 2004; Hunt et al., 2013; Heyman and Schaeken, 2015; Antoniou et al., 2016). In a picture-sentence verification study on *some* by Hunt et al. (2013), for example, among the 24 adult native speakers of English tested, 11 rejected over 80% of the underinformative sentences, while 10 accepted over 80% of the underinformative sentences and only 3 showed no strong preference. Researchers have begun to investigate what underlies this individual variation, examining which properties of the individual may modulate the extent to which they are likely to derive scalar implicatures during the processing of *some* in isolation (e.g., Nieuwland et al., 2010; Dieussaert et al., 2011; Marty and Chemla, 2013; Tomlinson et al., 2013; Heyman and Schaeken, 2015; Antoniou et al., 2016). We review this literature in Section “Two Accounts for Individual Differences in Scalar Implicature” below.

A handful of studies have investigated the effect of context on the comprehension of *some* by manipulating QUD within a discourse (e.g., Breheny et al., 2006; Zondervan et al., 2008; Politzer-Ahles and Fiorentino, 2013; Degen and Goodman, 2014; Dupuy et al., 2016; Politzer-Ahles and Husband, 2018). In a recent study on scalar implicature in French by Dupuy et al. (2016), participants were presented with visual stories in which a character acted upon all objects (e.g., a boy hiding five out of five car toys), and a question-answer dialog about the story between two puppets (Dupuy et al., 2016, experiment 3). The first puppet’s question was either “Did the boy hide all the cars?” or “Did the boy hide cars?”, representing either an upper-bound QUD or a lower-bound QUD, to which the second puppet answered, “The boy has hidden some cars.” Participants were asked to judge if the second puppet’s answer was right by selecting “yes” or “no.” Dupuy et al. (2016) found that participants were more likely to select “no” under the upper-bound QUD compared to the lower-bound QUD, suggesting that contextual information such as QUD does influence the comprehension of *some*. Although these studies have yielded evidence for the influence of context, none of them has systematically addressed individual differences. This leaves open the question of to what extent individuals differ in comprehending *some* utterances as dictated by the demands of context, and what abilities may make one better able to compute context-dependent interpretations for *some*. We address this question in the current study.

### Two Accounts for Individual Differences in Scalar Implicature

As discussed by Antoniou et al. (2016), the literature has yielded two main accounts which offer qualitatively different explanations for the individual variation observed in the comprehension of *some*: the “personality-based” account and the “cognitive resources” account.

The personality-based account posits that an individual’s likelihood of interpreting *some* with the *not all* implicature depends on personality traits, such as one’s awareness of the pragmatic use of language in everyday life (Nieuwland et al., 2010; Katsos and Bishop, 2011; Feeney and Bonnefon, 2013). Nieuwland et al. (2010), for example, examined the relationship between unimpaired adult individuals’ interpretation of *some* and their scores on the Autism-Spectrum Quotient (AQ, Baron-Cohen et al., 2001), a questionnaire assessing individuals’ autistic traits in a range of domains, including the everyday use of language in social communication (the Communication subscale of the AQ, “AQ-Comm subscale”). To examine individuals’ derivation of scalar implicatures, they compared brain responses to the object word in underinformative sentences (e.g., *lungs* in 6a) and in true and felicitous control sentences (e.g., *pets* in 6b).

(6a) Underinformative: Some people have lungs, which require good care.
(6b) True and informative: Some people have pets, which require good care.

Nieuwland et al. (2010) found a larger N400 EEG response for the object in underinformative sentences (6a) as compared to the control sentences (6b). However, this effect was limited to a subgroup of participants with better sensitivity to the pragmatic use of language in social communication as measured by AQ-Comm. In contrast, a subgroup with less sensitivity to the pragmatic use of language in social communication showed an effect in the opposite direction. Nieuwland et al. (2010) thus suggested that the ability to realize the *not all* implicature online depends on an individual’s awareness of the pragmatic aspects of language use in everyday life (see also Feeney and Bonnefon, 2013 for similar findings regarding the relation between self-perceived honesty and the scalar item *or*). However, the effects of personality-based factors have not been consistently found across studies; for example, Heyman and Schaeken (2015) did not find a robust relation between the interpretation of *some* and their personality-based measures, which included AQ and the Big-Five Personality Test, thus leaving open the question of to what extent personality traits modulate scalar implicature derivation.

In contrast to the personality-based account, the cognitive resources account proposes that variation in cognitive resources may affect the extent to which an individual is able to interpret *some* with the *not all* implicature. Scalar implicature has been characterized by some researchers as a costly process potentially involving multiple processing steps (De Neys and Schaeken, 2007; Huang and Snedeker, 2009; Dieussaert et al., 2011; Marty and Chemla, 2013; Tomlinson et al., 2013; see also Barbet and Thierry, 2016). For example, the generation of implicatures itself may be costly, which is particularly emphasized in psycholinguistic accounts of scalar implicature that reference processing cost in arguing that implicatures may be generated only when relevant to the context rather than by default. The interpretation of *some* also arguably involves processes such as the encoding and maintenance of information, including information regarding the context and the interlocutors in the context, in order to determine whether the interpretation of *some* is more optimal with or without the implicature. It may also require switching between generated interpretations of *some* that do or do not have implicature (or, under the Grammatical view, representations that do or do not include a silent operator), all of which may rely on an individual’s cognitive resources such as working memory and attentional control (see also Antoniou et al., 2016 for a recent discussion of how cognitive resources may come into play under alternative conceptions of how implicatures are generated).

Studies examining the influence of cognitive resources have typically adopted dual-task paradigms where participants respond to underinformative *some* statements while simultaneously attending to a secondary task to which cognitive resources must be allocated (De Neys and Schaeken, 2007; Dieussaert et al., 2011; Marty and Chemla, 2013; Heyman and Schaeken, 2015). Other studies have included independent measures of individuals’ cognitive abilities in order to test the relationship between these cognitive resources and the processing of *some* sentences (Antoniou et al., 2016). For example, Dieussaert et al. (2011) elicited participants’ true/false judgments for underinformative *some* sentences while simultaneously memorizing dot patterns. Participants were asked to first memorize a dot pattern. Next, they judged an underinformative *some* sentence. Finally, they were prompted to reproduce the dot pattern. Differing in complexity, the dot patterns were intended to engender either a high cognitive load or a low cognitive load. Individual participants’ working memory capacity was also assessed via the Operation Span Task. Dieussaert et al. (2011) found that participants were overall more likely to accept underinformative sentences when they tried to memorize high-load patterns than low-load patterns. This effect was only observed among the participants with low working memory capacity, while those with higher working memory capacity showed similar judgments regardless of high or low cognitive load. Dieussaert et al. (2011) thus suggested that realizing the *not all* implicature requires sufficient cognitive resources.

While the literature on scalar implicature has typically focused either on cognitive resources or on personality traits, to our knowledge there have only been a few studies on the derivation of scalar implicature for *some* that examined the role of both types of factor in the same study (Heyman and Schaeken, 2015; Antoniou et al., 2016; for an examination of scalar terms other than quantifiers, see Husband, 2014). Heyman and Schaeken (2015) examined the effect of a range of factors, including cognitive abilities and personality traits, on Dutch speakers’ judgments for underinformative statements based on world knowledge, such as *Some oaks are trees*. They found that neither cognitive nor personality-based factors robustly predicted individual variation in speakers’ judgments.

However, a recent study by Antoniou et al. (2016) revisited this issue, testing the effects of both types of individual differences on the interpretation of underinformative *some* sentences when underinformativity was established within the experiment, rather than based on world knowledge. In a picture-sentence verification task, participants were asked to judge underinformative statements like “There are hearts on some of the cards” as true or false, based on a visual display showing hearts on all five cards. Participants were also assessed on a battery of measures targeting cognitive resources and personality-based factors, including working memory (Backward Digit Span Task and Reading Span Task), attentional control (Stroop Task and the Simon task), cognitive flexibility (the Number-letter Task), autistic traits (Autism-Spectrum Quotient), personality traits (Big Five Inventory and Honesty/Integrity/Authenticity scale), and verbal and non-verbal IQ. Antoniou et al. (2016) found that interpreting *some* with the *not all* implicature was robustly predicted by working memory and age; individuals with larger working memory capacity were more likely to consistently derive the implicature (rejecting at least 4 out of 6 underinformative sentences in their study) than those with smaller working memory capacity, as were younger individuals. Other individual difference measures, including those assessing autistic and personality traits (e.g., Autism-Spectrum Quotient, Big-Five Inventory, and Honesty/Integrity/Authenticity Scale), did not turn out to be significant predictors. Antoniou et al. (2016) interpret the results as lending support for the cognitive resources account. They posit that the processes involved in computing scalar implicature may demand sufficient working memory, but also note that their stimuli, which included a high proportion of unambiguous fillers (e.g., when the picture shows 3/5 cards with stars), could also place a burden on working memory resources and thus hinder implicature generation. Regarding their finding that personality-based factors did not modulate scalar implicature derivation, Antoniou et al. (2016) suggest that personality-based factors might not account for variability in interpreting *some* robustly when working memory is included in the analysis, which previous studies like Nieuwland et al. (2010) did not test. However, they also discuss the possibility that personality-based factors may be more important for interpreting *some* sentences in richer discourse contexts. They speculate that, when tested in more naturalistic/communicative discourse contexts, it is possible that both cognitive and personality-based factors may modulate an individual’s likelihood of deriving the implicature for *some* (see Marty and Chemla, 2013 for similar discussion).

## Current Study

In the current study, we examine the role of individual differences in the derivation of scalar implicatures for *some* when presented in a communicative discourse context involving two interlocutors. Our primary aim is to directly test whether individual differences in scalar implicature derivation for *some* in context are better accounted for by cognitive resources or by personality-based pragmatic abilities, or whether both cognitive and personality-based abilities may play a role, as speculated by Antoniou et al. (2016). We address the following two main research questions in this study: First, we examine whether individuals’ interpretation of *some* is influenced by the QUD, which is established via a brief discourse context involving utterances by two interlocutors. If comprehenders are able to utilize QUD in interpreting *some* in context, then the *not all* implicature should be more likely to arise when the QUD makes it relevant (upper-bound) than when the QUD does not (lower-bound). Second, we examine which properties of the individual modulate one’s ability to interpret *some* based on the QUD, by including a battery of measures targeting abilities that are potentially important for computing a context-dependent interpretation of *some*, including measures targeting both cognitive resources and personality-based pragmatic abilities. We test the prediction that not only cognitive resources but also personality-based pragmatic abilities may affect individuals’ ability to utilize QUD in interpreting *some*, given that the current study examines scalar implicature derivation in a discourse context involving communication between two interlocutors. As we discuss below, we also include assessments of individuals’ language abilities to address to what extent language abilities may account for variation in scalar implicature derivation in context.

To address these questions, we tested participants using a story-sentence matching task in which one character utters a sentence containing *some*, such as “I folded some of the sweaters,” following a question from another character that either establishes the relevance of the *not all* implicature (e.g., “Did you fold all the sweaters?”) or that the implicature is irrelevant (e.g., “Did you fold any sweaters?)”. How many objects were acted upon is illustrated in a visual display (showing, e.g., either 0, 2, or 4 folded sweaters). If an individual is sensitive to the context (whether the implicature has been established as relevant or as irrelevant), then they should be less likely to accept the target sentence (e.g., “I folded some of the sweaters,” when 4 of 4 sweaters had been folded) when the implicature is relevant than when it is irrelevant.

Participants also completed a battery of individual differences assessments targeting cognitive resources and personality-based pragmatic abilities, allowing us to directly test proposals that individual differences in scalar implicature derivation have their origin in either cognitive resources or personality-based pragmatic abilities, or instead may make recourse to both types of ability. In addition to examining these two commonly tested potential sources of variation (cognitive resources and personality traits), we also examine individual differences in language skills as a third possible source of variability. Relationships between language skills and pragmatic abilities have been explored in studies in the literature on children (e.g., Katsos et al., 2011) and on adults with Autism and Asperger Syndrome (e.g., Pijnacker et al., 2009). Yet in studies examining unimpaired adults, language skills have rarely been tested in the scalar implicature literature as a possible source of individual variability. Therefore, the current study included language skills as a third measure of individual differences, in order to examine whether language skills may be among the sources of variability contributing to individual differences in scalar implicature derivation among unimpaired adult native speakers.

### Participants

Sixty-four native English speakers (19 male, mean age = 21.9, age range = 18–53) who were naïve about the purpose of the study were recruited from the University of Kansas community. All participants provided informed consent before participating and received a cash payment or course credit upon completing their visit.

### Main Task: Story-Sentence Matching Task

**FIGURE 1:**
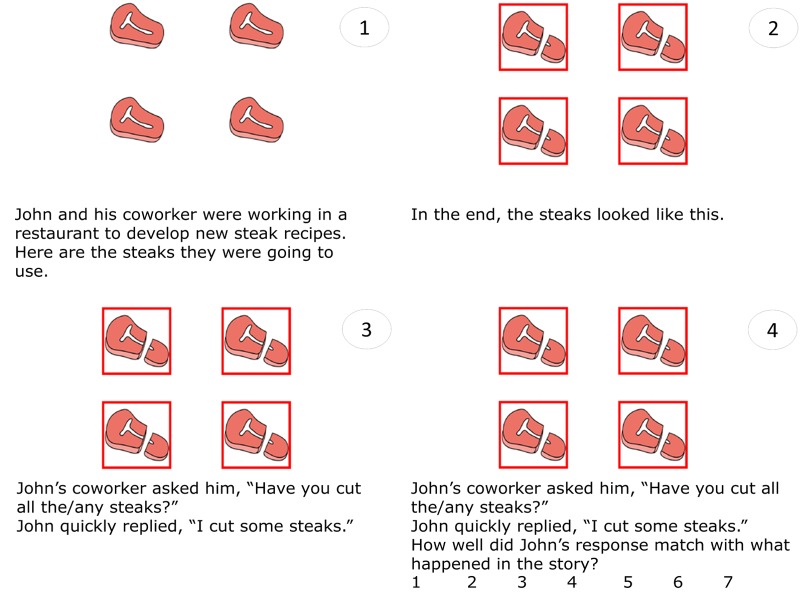
Sample display of a target trial in the main story-sentence matching task. Each trial is depicted on four consecutive slides, numbered 1–4 here.

The current study utilized a story-sentence matching task to probe the interpretation of *some* in a context in which the interpretation of the target *some* sentences depends on the QUD. We constructed 32 target trials, each of which consisted of a short story presented in text and pictures on a series of slides, about two characters carrying out an action involving a set of four objects (e.g., cutting four pieces of steak). The first slide introduced the characters and the objects. The second slide always showed that 4/4 objects were changed (e.g., all four steaks were shown as cut); following Hunt et al. (2013), all acted-upon objects were also highlighted by a red square to remove any ambiguity regarding the number of objects acted-upon, and the text showed that the objects ended up as shown in the picture (e.g., “In the end, the steaks look like this.”). On the third slide, a brief conversation between the characters was presented, in which one character asked, “Have you cut all the steaks?” or “Have you cut any steaks?” and the other responded “I cut some steaks.” Then a rating question appeared, asking the participant to rate how well the response matched with what happened in the story on a 7-point Likert scale. After they responded by clicking on their chosen rating on the scale, a button showing “click here for the next story” appeared at the bottom center of the screen, which triggered the next story once clicked on (see **Figure 1** for the depiction of an example trial). The slides were automatically presented at a comfortable reading speed (first slide 8000 ms, second slide 3000 ms, third slide 4000 ms), and the rating question and the scale were untimed. Participants were asked to read the stories carefully and answer the question at the end of each story.

In the target trials, we established an upper-bound or lower-bound QUD by including “all” or “any” in the first character’s question sentence, following Politzer-Ahles and Fiorentino (2013). When the question includes “all,” as in “Have you cut all the steaks?”, the QUD is established as upper-bound, as the character is asking about whether each and every steak in the set has been cut. Therefore, the response sentence with *some* is expected to be interpreted as *at least one but not all*, such that the added *not all* implicature addresses the upper-bound QUD. The response should thus be underinformative since all four objects were acted upon as shown by the picture. In contrast, when the question includes “any,” as in “Have you cut any steaks?”, the QUD is established as lower-bound, as the character is asking about whether at least one steak has been cut. In this scenario, *some* is expected to be interpreted as *at least one* without the *not all* implicature. Thus, the response sentence with *some* should be felicitous given that at least one object has been acted upon. Therefore, if comprehenders are sensitive to the QUD in interpreting *some*, they should rate the target *some* sentences lower when the question sentence includes “all” (the upper-bound QUD; *all* condition, henceforth), compared to when the question sentence includes “any” (the lower-bound QUD; *any* condition, henceforth).

In order to mask the purpose of the study and to elicit participants’ interpretation of *some* in unambiguously true/felicitous or false sentences, we also included 32 filler trials that have the same story structure, presentation format, and QUD manipulation as the target trials. However, the filler trials differed from the target trials in two ways: (1) the character’s answer sentence included *only some* instead of *some* (e.g., “I cut only some steaks”), which should be unambiguously interpreted as *some but not all*; and (2) the number of acted-upon objects in the picture was 0/4, 2/4 or 4/4, while there were always 4/4 acted-upon objects in the target trials. These configurations thus made the filler trials patently true or patently false depending on the number of acted-upon objects, regardless of the QUD being upper-bound or lower-bound; the fillers were true when 2/4 objects were acted upon, and false when either 0/4 or 4/4 objects were acted upon.

From the two target conditions (*all* condition and *any* condition), we generated two lists of targets by alternating the QUD for each story, such that each participant would see all 32 target stories, but no participant would encounter the same story in both conditions. Each list included a total of 64 unique stories presented in random order (32 targets and 32 fillers), with half of the targets (16) in the *all* condition and the other half (16) in the *any* condition. Participants were randomly assigned to complete only one list. The truth value of the response sentences, number of acted-upon objects, and the QUD were balanced across all the trials (see **Supplementary Table S1**, for a summary of the properties of the target and filler stimuli).

### Measures of Individual Differences

The current study examined three potential sources of individual variation as discussed above (cognitive resources, personality-based socio-pragmatic abilities, and language skills), testing participants on a battery of measures targeting these three domains. In the following section, we describe how these sources of variations were assessed.

#### Measures of Cognitive Resources

##### Working memory capacity: count span task

Working memory capacity has been widely suggested as a factor that may account for individual differences in deriving scalar implicatures (De Neys and Schaeken, 2007; Dieussaert et al., 2011; Marty and Chemla, 2013). When interpreting *some* under a specific QUD in the discourse, as is required in the current study, sufficient working memory may also be required to encode and maintain the QUD throughout the task, and to accurately represent what happens in an event which spans across multiple sentences and visual representations of the story scene. Thus, interpreting *some* in context and making distinctions between QUDs may require sufficient working memory capacity.

In the current study, we assessed individual working memory capacity via Count Span (Conway et al., 2005), which measures non-verbal working memory in a counting and recalling task. In the Count Span task, the participant was asked to count out loud the number of appearances of a specific shape when they viewed an array of shapes on the computer screen. The experimenter recorded the numbers that the participant had counted on each screen, after which a screen with a new array of shapes appeared. After between 2 and 6 screens, the participant would be prompted to recall the numbers they counted on the previous set of screens in their order of occurrence by entering the digits on the keyboard. Following Conway et al. (2005), we calculated an accuracy score for this task by comparing their total number of correctly recalled digits versus the total numbers of counted digits.

##### Domain-general context maintenance: dot pattern expectancy task

Although the previous literature has tended to focus on working memory as a factor that may modulate an individual’s ability to process scalar implicatures, two additional processing-related factors which may be particularly important in processing *some* in context are domain-general context-maintenance ability and attentional control. Domain-general context maintenance involves holding prior information in working memory and utilizing it to subsequently determine task-relevant responses (Cohen et al., 1999). This ability has been examined as a potentially important source of variability in studies on the influence of context on ambiguity resolution in language tasks (e.g., Cohen et al., 1999, lexical ambiguity; Boudewyn et al., 2015, referential ambiguity). The ability is arguably also relevant when processing *some* under different QUDs, which involves maintaining the prior cues to the QUD and using them to determine whether an upper-bound or lower-bound meaning is supported under the current context. Therefore, distinguishing between QUDs and utilizing them in the interpretation of *some* sentences may require sufficient context maintenance ability.

The current study assessed domain-general context maintenance ability via the Dot Pattern Expectancy Task (DPX), a version of the Continuous Performance Test in which participants respond to visually presented cue-target pairs, and must make a designated response only when both the cue and the target come in a specified form (Rosvold et al., 1956; Cohen et al., 1999). In the DPX, each trial includes a pair of dot patterns, with a “cue” pattern in white and a “probe” pattern in blue. The trials were comprised of four types: AX, AY, BX, and BY. AX trials are the target trials, while all other types are non-targets in that they either include a non-target probe pattern (AY), or include a non-target cue pattern followed by a target or non-target probe pattern (BX and BY, respectively). Therefore, the identity of the cue determines whether the following probe constitutes a target trial, as a non-target cue directly indicates a non-target trial regardless of the identity of the probe. For an individual with a high level of context maintenance ability, they should be able to not only correctly recognize the target pattern for AX trials, but also correctly detect the non-target cue (context) and refrain from making a target response for BX trials despite the target probe. In contrast, an individual with lower context maintenance ability should make more errors in BX trials by ignoring the context and incorrectly making a target response only based on the probe.

When completing this task, participants were instructed to press either a “no” or a “yes” button on a keyboard upon seeing each dot pattern; they should only press “yes” after seeing the target blue pattern following the target white pattern, and press “no” for all the other patterns. After each key press, they heard either a “bing” or a “buzz” sound indicating whether they had pressed the correct key. Four practice sessions were administered at the beginning of the task along with instructions. Participants practiced until they had reached 80% accuracy and had responded correctly to at least 1 BX trial, before they began the main session. The task was administered using Paradigm (Tagliaferri, 2005), with a cue duration of 1000 ms, an interstimulus interval of 2000 ms, target presentation for 500 ms, a response window of 1500 ms, and an intertrial interval of 1200 ms. There were 144 trials in total (104 AX, 16 AY, 16 BX, and 8 BY), with each trial type evenly distributed across four blocks.

A *d*-prime score was computed for the DPX task, following Cohen et al. (1999). The *d*-prime indexes the sensitivity to context in this task, which accounts for accuracy including both hit rate on target trials (AX trials) and false-alarms (BX trials, which included non-target cue pattern followed by a target). For each participant, their *d*-prime was calculated by *z*(the accuracy on AX trials) -*z*(the error rate on BX trials). Following standard procedure, hit rates of 1 were corrected to (1 - 1/160), and false alarm rates of 0 were corrected to 1/16. Higher *d*-prime scores represent greater sensitivity to domain-general context cues.

##### Attentional control: number Stroop task

Attentional control involves the ability to attend to crucial information in the presence of distractions and to inhibit the information that is irrelevant to the current task (e.g., Kane and Engle, 2002). Higher levels of attentional control ability have been found to facilitate performance in cognitive and language tasks involving selective attention and suppression of irrelevant information (e.g., Hutchison, 2007; Bialystok and Martin, 2004; Boudewyn et al., 2012; Abutalebi and Green, 2016). In the literature on scalar implicature, a handful of studies have included measures of attentional control or inhibition ability as a submeasure of cognitive resources involved in interpreting *some* in isolation, although they have not commonly found it to have a significant effect (e.g., Heyman and Schaeken, 2015; Antoniou et al., 2016). We included an attentional control measure in the current study since sufficient attentional control may be important for generating QUD-dependent interpretations for *some* in context, where the comprehender needs to suppress one interpretation and pursue the other one that is relevant under the current QUD, while processing a relatively large amount of linguistic and visual material compared to a typical study on *some* in isolation.

We assessed attentional control via the number Stroop task, following the procedure outlined in Bush et al. (2006). In each trial, the participant was asked to count the number of words presented on the computer screen, which could be any number between 1 and 4, and to press the corresponding number key as quickly and as accurately as possible on a button pad. Trials were presented in 8 blocks of 20 trials; 4 blocks included congruent trials and 4 included incongruent trials, the order of which was counterbalanced across blocks. In congruent trials, the words were common animal words (e.g., “dog dog”; correct response is 2), while in incongruent trials the words were number words that do not match with the quantity of words on the screen (e.g., “one one one”; correct response is 3). Thus, for incongruent trials, participants must maintain their attention toward the quantity of words on the screen while avoiding distraction from the meanings of the words, in order to achieve the correct answer. We computed a Stroop interference score for each participant by subtracting the percent accuracy for congruent trials from that of incongruent trials, such that higher values reflect better attentional control ability^2^.

#### Personality-Based Socio-Pragmatic Abilities

A number of studies have suggested that individual variation in the derivation of scalar implicature has its origin in personality traits (Nieuwland et al., 2010; Katsos and Bishop, 2011; Feeney and Bonnefon, 2013). As Antoniou et al. (2016) speculate, personality-based factors such as sensitivity to the pragmatic use of language in everyday life may be particularly important when processing language in more conversational settings, as opposed to when interpreting *some* sentences outside of any discourse. Thus, an individual’s ability to utilize QUD in order to arrive at a pragmatically felicitous interpretation of *some* in context in the current study may depend at least in part on their sensitivity to the pragmatic use of language in everyday life (which we refer to below as their socio-pragmatic abilities).

In the current study, socio-pragmatic abilities were assessed via Autism-Spectrum Quotient questionnaire (AQ, Baron-Cohen et al., 2001), which assesses individuals’ general social and communicative skills based on the level of autistic-like traits that their responses demonstrate. The questionnaire includes 50 statements about self-perceived characteristics, with 10 statements from each of the five subscales examining traits that vary across the autism spectrum (social skills, attention switch, communication, attention to detail, and imagination). Participants were asked to read each statement and answer to what degree each statement truly reflects themselves, by choosing from four levels: definitely agree, slightly agree, slightly disagree, or definitely disagree. Following Baron-Cohen et al. (2001), the answers were scored by assigning 1 to “definitely agree” or “slightly agree,” and 0 to “definitely disagree” or “slightly disagree” for the statements that indicate strong autistic traits, and assigning 0 to “definitely agree” or “slightly agree,” and 1 to “definitely disagree” or “slightly disagree” for the statements that do not indicate strong autistic traits. The total score and the scores for each of the five subscales were calculated by adding up the scores for the corresponding items. Thus, higher AQ scores are taken to reflect weaker socio-pragmatic abilities.

#### Language Skills

A third source of variation that may modulate an individual’s derivation of scalar implicatures is language skills. Although native speakers have been assumed to share a native grammar and thus have similar language abilities, recent studies have revealed that adult monolingual native speakers do show variability in native language processing (e.g., Kemper and Sumner, 2001; Pakulak and Neville, 2010; Borovsky et al., 2012; Dąbrowska, 2012; Van Dyke et al., 2014). Language skills have been shown to play an independent role in accounting for variability in native language processing in a range of domains, even when examined together with assessments of non-linguistic cognitive resources such as working memory (Van Dyke et al., 2014), which recommends the inclusion of language skills in studies examining individual variation in studies on language comprehension.

The measures of language skills in the current study targeted vocabulary, assessed by the Peabody Picture Vocabulary Test 4th edition (PPVT-4, Dunn and Dunn, 2007), and exposure to print materials, assessed by Author and Magazine Recognition task (Acheson et al., 2008). Vocabulary skills have been shown to predict comprehension success at the word level and the sentence level (e.g., Braze et al., 2007; Perfetti, 2007; Boudewyn, 2015). It has been argued that having strong, detailed lexical representations leads to efficient and successful comprehension in a number of ways, such as by reducing interference between lexical representations during comprehension (see e.g., Van Dyke et al., 2014 for recent evidence). It has also been suggested that that those with stronger lexical representations may be better able to process the meanings of words and integrate words in context in order to derive meanings and generate inferences during passage comprehension (see e.g., Hamilton et al., 2013). When interpreting *some* in context, those with better vocabulary skills may be better able to recognize the two possible readings of *some* and to select an optimal reading according to the current context.

Print exposure has been shown to account for individual differences in performance across several linguistic domains, ranging from orthographic and phonological processing through sentence and discourse comprehension (Acheson et al., 2008; Arnold et al., 2018). It has been argued that increased print exposure may lead to increased sensitivity to linguistic cues that facilitate comprehension, including as regards the resolution of ambiguity in discourse (e.g., Arnold et al., 2018). Arnold et al. (2018) found that individuals with greater print exposure as assessed by the Author and Magazine Recognition Task were better at resolving ambiguous pronoun reference using discourse cues. Increased sensitivity to discourse cues and patterns as a result of greater print exposure may also impact the interpretation of *some* in context; individuals with greater print exposure may be better able to recognize and utilize QUD in order to arrive at a coherent interpretation of the ambiguous term *some* in the discourse.

##### Vocabulary: Peabody Picture Vocabulary Test

The PPVT-4 is a standardized test of receptive vocabulary that spans several subject fields. In each trial, the participant heard an English word pronounced by an experimenter and was asked to select from among four pictures the one that corresponds to the word. The trials were numbered and organized into sets of 12, with increasing level of difficulty. A starting set was initially picked based on the participant’s chronological age, following the PPVT-4 manual. If the participant made 2 or more errors in this set, then the experimenter would go back to the previous set and test that as the new starting set, until the participant made 1 or 0 errors in a set. As they responded to the trials, the participant’s answers were manually recorded and the total number of errors within each set was tracked by the experimenter on the PPVT-4 testing booklet. The task came to an end either when the participant made 8 or more errors within a set, or when they have completed the last set of the entire test. For each participant, a raw score was first computed by subtracting the total number of errors from the number of completed items; this raw score was then standardized based on the participant’s chronological age, using the standardization chart provided in the PPVT-4 manual.

##### Exposure to print materials: Author and Magazine Recognition Task

We measured exposure to print materials via the Author and Magazine Recognition Task (ART and MRT, Acheson et al., 2008). The ART consists of a list of 130 author names and the MRT a list of 130 magazine titles. Half of the names in the ART are real authors’ names and the other half are foils that look like author names; similarly, half the titles in the MRT are real magazine titles and the other half are foils that appear to be magazine titles. The real items in both tasks are from popular reading materials covering various topics and genres. Participants were asked to select real authors’ names in the ART and real magazine titles in the MRT without guessing, by entering an “X” beside the items in an Excel spreadsheet. Following Acheson et al. (2008), answers were scored by assigning 1 point for a correctly identified real item, and -1 point for a foil item incorrectly identified as real, generating a total score for the ART and for the MRT for each participant. The ART and MRT scores were then averaged into one single score, with higher values reflecting more extensive print exposure.

### Composite Scores for Measures of Individual Differences

We computed composite scores for cognitive resources and for language skills, based on the conceptual relatedness of the specific measures and the correlations among the scores within each domain (see **Supplementary Table S2**, for summary statistics for each of the individual difference measures, and **Supplementary Table S3** for pairwise correlations between the measures). That is, the composite score for Cognitive Resources was calculated by summing the Count Span score, Dot-pattern Expectancy *d*-prime score, and Stroop interference score. The Language Skills composite was calculated by summing the standardized PPVT-4 score and the Author and Magazine Recognition task score. Total AQ score was used to quantify individuals’ Socio-pragmatic Abilities. We used Total AQ rather than the AQ-Communication Subscale (used, e.g., in Nieuwland et al., 2010), since all the subscale scores strongly correlated with the total AQ score. However, we note that the pattern of results reported below does not change if the AQ-Comm score rather than Total AQ is used in the analysis. This generates three individual difference scores that were included as predictors in the model-fitting: Cognitive Resources, Socio-pragmatic Abilities (Total AQ score), and Language Skills^3^. Before model fitting, the three scores were standardized using *z*-transformation, so that they are on similar numerical scales as required by mixed effect models (Jaeger, 2008).

### Procedure

Participants completed all the experimental tasks in the Neurolinguistics and Language Processing Laboratory at the University of Kansas. Tasks were administered in the following order, with break times in between each task: Peabody Picture Vocabulary Test 4th edition, Autism-Spectrum Quotient questionnaire, Author and Magazine Recognition Task, the Story-sentence Matching Task, Count Span task, Dot Pattern Expectancy task, and Number Stroop task. The entire session took about 1 h 30 min to complete in one visit to the laboratory.

### Summary of Predictions

Our first research question concerns the extent to which participants are sensitive to QUD in interpreting *some*. If participants are able to utilize QUD in interpreting *some*, then a main effect of QUD is expected to emerge, such that the ratings for the target sentences should be lower in the *all* condition than those in the *any* condition. Our second research question concerns the role of individual differences in the context-sensitive interpretation of *some*. If the interpretation of *some* in context is impacted by individual differences in both Cognitive Resources and Socio-pragmatic Abilities, then we expect interactions to emerge between QUD and the Cognitive Resources composite measure, as well as between QUD and the Socio-pragmatic Abilities measure. If individual differences in Language Skills also impact the interpretation of *some* in context, an interaction between QUD and the Language Skills composite measure is expected to emerge.

## Results

### Data Pre-processing and Modeling

The ratings in the main experiment were statistically analyzed by fitting a cumulative link mixed model (the *clmm* function from the package *ordinal*) with a *probit* link function (Christensen, 2010) in the R programming environment. We chose to use the cumulative link mixed model as it can analyze categorical outcomes while incorporating subject-level and item-level random effect structures, which is an advantage over traditional regression models (Jaeger, 2008; Cunnings, 2012). The *probit* link function allows us to analyze rating responses by underlyingly modeling the log-transformed odds ratio of increasing the rating by 1 on the Likert scale (e.g., rating an utterance as 5 over 4, or as 6 over 5, etc., on the 7-point scale).

Model fitting began by including the following predictors of interest: the fixed factors QUD (*all*, *any*), and interactions between QUD and each of the individual difference scores: QUD × Cognitive Resources, QUD × Socio-pragmatic Abilities, and QUD × Language Skills. Participant and Item were included as random intercepts. The initial model was then optimized by backward-fitting via log-likelihood ratio tests: if removing a predictor from the initial model did not reduce the model fit, then a simpler model without that predictor was built; on the contrary, if removing a predictor led to worse fit, then the predictor was retained. Following this procedure, the final model included the fixed effect of QUD and two interaction terms: QUD × Cognitive Resources, QUD × Socio-pragmatic Abilities, as well as Participant and Item as random intercepts.

### Effects of QUD and Individual Difference Measures

The two main research questions in the current study concern whether QUD modulates the rating of *some* sentences, which should be reflected by lower ratings in the *all* condition than in the *any* condition, and to what extent sensitivity to QUD is subject to individual differences in cognitive resources, personality-based pragmatic abilities, and language abilities, which would be reflected in a significant interaction between the QUD and a given measure of individual differences. Although all the variables of interest for both research questions were incorporated into one model, we report the results separately for each research question below.

A few things should be kept in mind when interpreting the effects in the final model, which is summarized in **Table 1**. Because of the *probit* link function, the coefficients represent the effect of predictors on the odds ratio of increasing the ratings, not directly on the ratings *per se*. Regarding the QUD effect, as the *all* condition was dummy-coded as the baseline condition, the effect of QUD appeared as the effect of the *any* condition, as compared to the *all* condition. Finally, since the individual difference scores have been standardized to fit in the same model, the effects involving these scores should be interpreted based on standardized units.

**Table 1:** Summary of the final model analyzing *N* = 64 participants’ ratings as a function of QUD and individual difference measures.

	β	*SE*	*z*	*p*
QUD	0.5963	0.0487	12.25	<0.001
QUD × Cognitive Resources	0.13935	0.04896	2.846	<0.01
QUD × Socio-pragmatic Abilities	–0.19605	0.04774	–4.107	<0.001


*QUD reflects the difference between the ratings in the any condition compared to the all condition, which is coded as the baseline.*

To address the role of QUD, we examined the main effect of QUD in the model. The main effect of QUD is indeed significant, indicating that overall participants were more likely to provide higher ratings for target utterances in the *any* condition compared to the *all* condition (β = 0.5963, *SE* = 0.0487, *z* = 12.25, *p* < 0.001). In short, this finding suggests that the derivation of the scalar implicature for *some* was affected by the QUD as established in the discourse context (see **Supplementary Figure S1** for a visualization of the differences in mean raw ratings between the *all* condition and the *any* condition). To confirm that this effect of QUD does not just reflect an overall preference for the *any* versus the *all* stimuli regardless of whether the stimuli contained *some* (the targets, where QUD matters) or *only some* (the fillers, where QUD does not matter), we examined responses to the fillers, which also had *all* versus *any* QUDs but had a target sentence with *only some*, where ratings should not be sensitive to QUD. As expected, QUD did not modulate ratings in the fillers (β = 0.1334, *SE* = 0.8479, *z* = 0.157, *p* = 0.875).

To address whether sensitivity to QUD in interpreting *some* is impacted by individual differences in cognitive resources, personality-based pragmatic abilities, and language skills, we examined interactions between the QUD effect and individual difference scores in each of these three domains. Among the individual difference measures, QUD significantly interacted with both Cognitive Resources (β = 0.1394, *SE* = 0.0489, *z* = 2.846, *p* < 0.05) and Socio-pragmatic Abilities (β = -0.1961, *SE* = 0.0477, *z* = -4.11, *p* < 0.001), indicating that the QUD effect is modulated by individual differences in both domains. Sensitivity to QUD increased with greater cognitive resources, and with better socio-pragmatic abilities (note that since better socio-pragmatic abilities are indexed by lower AQ scores, the coefficient for the interaction term QUD x Socio-pragmatic Abilities is negative). Regarding the role of Language Skills, the fact that QUD × Language Skills was excluded during the model fitting indicated that Language Skills was not a significant predictor of individual sensitivity to QUD in our study.

## Discussion

The current study investigated the interpretation of the scalar quantifier *some* in contexts which establish the *not all* scalar implicature as relevant (upper-bound contexts) or irrelevant (lower-bound contexts). We examined to what extent native speakers are sensitive to context in interpreting *some* and which individual differences may best account for variability across individuals in the ability to utilize contextual information to interpret *some*. Overall, we found that native speakers as a group do distinguish the meaning of *some* based on the QUD, such that the *not all* implicature is more likely to arise under an upper-bound QUD than a lower-bound QUD. While the interpretation of *some* is typically described as context-sensitive in linguistic analyses, the findings of the current study converge with those of a still relatively limited number of experimental studies in demonstrating sensitivity to QUD in the interpretation of *some* during language comprehension (Politzer-Ahles and Fiorentino, 2013; Degen and Goodman, 2014; Dupuy et al., 2016; Politzer-Ahles and Husband, 2018). However, the findings of the current study also revealed individual differences in the extent to which QUD affects the interpretation of *some*, which depended both on an individual’s cognitive resources and on their personality-based pragmatic abilities. While previous studies on the processing of *some* in isolation have alternatively argued that the derivation of scalar implicatures depends on cognitive resources or on personality traits, our findings are unique in demonstrating that the derivation of scalar implicatures, when tested in a discourse context, indeed makes recourse to both types of abilities.

### The Role of Cognitive Resources in Context Sensitivity

Our finding that individuals with greater cognitive resources show greater sensitivity to the context in interpreting *some*, as evidenced by the significant interaction of QUD × Cognitive Resources, converges with the those of a number of studies arguing that sufficient cognitive resources are required for an individual to derive scalar implicatures (e.g., De Neys and Schaeken, 2007; Dieussaert et al., 2011; Marty and Chemla, 2013). In our study, there are a number of possible ways that greater cognitive resources may have led to increased sensitivity to QUD. The interpretation of *some* with respect to a given QUD requires successfully attending to the contextual cues that establish QUD, as well as the encoding and maintenance of that information throughout the discourse. Upon encountering *some*, previously encountered information needs to be recalled and utilized to compute a context-sensitive interpretation for *some*, and the selected meaning for *some* must be maintained while possibly inhibiting the other meaning. All of these processes would arguably make recourse to the kinds of cognitive resources assessed in the current study (working memory, attentional control, and ability to maintain contextual information during processing), which regard an individual’s ability to encode and maintain information and direct attention while also processing bottom-up input. Individuals with greater cognitive resources may also be better at consistently attending to and utilizing contextual information in order to interpret the target utterances over the course of an experiment that involved a relatively large number of target and filler trials, which itself may incur some amount of processing burden.

### The Role of Personality-Based Factors in Context Sensitivity

The current study also revealed that personality-based factors such as socio-pragmatic abilities (as measured by the AQ) also modulated sensitivity to QUD; those with greater socio-pragmatic abilities made a larger distinction between QUDs in their ratings, thus lending support to accounts arguing that personality traits modulate an individual’s likelihood of deriving scalar implicatures (e.g., Nieuwland et al., 2010; Feeney and Bonnefon, 2013). In the current study, those individuals with greater awareness of the pragmatic aspects of communication in daily life, as assessed by AQ, were more sensitive to whether the *not all* implicature for *some* had been established as relevant within the conversational context.

Our findings converge with a number of previous studies demonstrating relationships between scalar implicature derivation and cognitive resources on one hand, and with a number of studies demonstrating relationships between scalar implicature derivation and personality traits on the other hand. Interestingly, in one previous study on the derivation of scalar implicature for *some* in isolation which did assess both potential sources, personality-based factors were not found to be a significant predictor of realizing the *not all* implicature (Antoniou et al., 2016). Recall that Antoniou et al. (2016) examined the interpretation of *some* without discourse context, where the acceptability of *some* sentences should only depend on a visual depiction that either made them felicitous or infelicitous. As Antoniou et al. (2016) acknowledged, socio-pragmatic abilities may not robustly modulate the interpretation of *some* in this type of task as it does not establish any kind of conversational exchange or discourse involving more than one interlocutor, and thus may not prompt the participant to make use of their understanding of the pragmatics of conversation in deciding how to interpret *some*.

The fact that Antoniou et al. (2016) did not observe an effect of personality-based pragmatic abilities while the current study did find such an effect is consistent with the claim that these abilities may be particularly important for taking contextual information into account when interpreting *some*, in particular that from communicative discourse contexts. This is exactly the kind of context provided in our story-sentence matching task, where a conversation between two interlocutors established the QUD determining the relevance of the implicature. Our findings thus strongly argue that individuals rely on both types of ability in the interpretation of *some* under conversational discourse contexts.

### The Role of Language Skills

Among our individual difference measures, language skills (measured via a composite of vocabulary size and exposure to print materials) did not prove to modulate individual sensitivity to QUD in interpreting *some*. It is worth noting that in Antoniou et al. (2016), their measure of verbal IQ (a sentence repetition task) also did not significantly predict individuals’ derivation of the *not all* implicature. Although neither the current study nor Antoniou et al. (2016) found evidence of a relationship between language skills and implicature derivation for *some*, a question to be examined in future research is whether language skills may become increasingly important when the relevance of the implicature is established in more linguistically rich contexts, perhaps with less visual information, which may place greater demands on the comprehender to construct and process the discourse through careful comprehension of a larger amount of text or speech input. Future studies could also examine whether different measures of language abilities might better account for individual variability in the derivation of scalar implicatures in context, such as passage comprehension measures which more directly target the processing of discourse.

More broadly, it may also be interesting for future research to examine to what extent language skills as well as cognitive resources and personality-based factors may influence the derivation of implicatures for scalar terms other than the quantifier *some*. Moreover, future research examining individual differences in sensitivity to context in the interpretation of scalar terms using online measures such as self-paced reading (e.g., Breheny et al., 2006; Politzer-Ahles and Fiorentino, 2013), eye-tracking (e.g., Politzer-Ahles and Husband, 2018), or neurolinguistic methods (e.g., Hartshorne et al., 2015; Politzer-Ahles and Gwilliams, 2015), may also provide new insights regarding how individual differences in the domains examined in the current study impact the derivation of scalar implicatures during the dynamics of language processing.

## Conclusion

This study demonstrates that comprehenders vary in their ability to utilize context cues in interpreting *some* in context. Moreover, this variability is associated with individual differences in both cognitive resources and personality-based pragmatic abilities. While previous studies on the processing of *some* without manipulating context have argued for one or the other of these sources in order to account for individual variability in deriving scalar implicatures, the current study establishes for the first time that computing pragmatically enriched meanings based on the broader discourse indeed draws upon both kinds of skills.

## Ethics Statement

This study was carried out in accordance with the recommendations of the Human Research Protection Program (HRPP) at the University of Kansas. The protocol was approved by the Institutional Research Board at the University of Kansas (Study #00004409). All subjects gave written informed consent in accordance with the Declaration of Helsinki.

## Data Availability

The de-identified raw data supporting the conclusion of this manuscript will be made available by the authors, without undue reservation, to any qualified researcher upon written request.

## Author Contributions

XY, UM, and RF contributed the conception and design of the study. XY administered the experiment, organized the data, and performed the statistical analyses. XY, UM, and RF contributed to manuscript writing and editing.

## Conflict of Interest Statement

The authors declare that the research was conducted in the absence of any commercial or financial relationships that could be construed as a potential conflict of interest.

## Footnotes

1 For example, under the Default view (Levinson, 2000), the implicature can be canceled when it is irrelevant in the context, while under Relevance Theory view (Sperber and Wilson, 2002), context may affect whether the implicature is generated in the first place (see also the constraint-based account recently proposed by Degen and Tanenhaus, 2015). Under the Grammatical view (Chierchia, 2004), one may argue that context determines whether the silent operator is present or not. As such, the context manipulation in the current study is not intended to adjudicate among these alternative accounts of scalar implicature derivation, but instead to examine to what extent comprehenders are indeed sensitive to contextual information when interpreting *some*, and to probe the extent and origins of individual differences in sensitivity to context in the interpretation of *some*.

2 We also computed Stroop interference scores based on reaction time (by subtracting the reaction time of incongruent trials from congruent trials); since this score was correlated with the accuracy-based interference score (*r* = 0.316), only the accuracy-based interference score is used in the analysis.

3 We note that the composite score for Cognitive Resources, the composite score for Language Skills, and the Socio-Pragmatic Abilities score are not correlated (see **Supplementary Table S4** for pairwise correlations between the composite scores).
