# Supplementary material for: Context-Sensitivity and Individual Differences in the Derivation of Scalar Implicature
Source: Front Psychol. 2018 Sep 20;9:1720. doi: 10.3389/fpsyg.2018.01720 (PMC6158351; doi:10.3389/fpsyg.2018.01720)
Supplement: Supplementary file 1 [file Table_1.pdf]

Supplementary Material

Context-sensitivity and Individual Differences in the Derivation of Scalar Implicature

Xiao Yang*, Utako Minai, Robert Fiorentino

*** Correspondence:** Xiao Yang: xiaoyang@ku.edu

Supplementary Tables

Table 1: A summary of trial types and properties in the main story-sentence matching task.

<table>
<tr><th></th><th>No. of trials</th><th>QUD</th><th>Quantifier in the target utterance</th><th>No. of objects changed in the picture</th><th>Felicity and truth value</th></tr>
<tr><td rowspan="2">Targets</td><td>16</td><td>All</td><td>some</td><td>4 out of 4</td><td>True but infelicitous</td></tr>
<tr><td>16</td><td>Any</td><td>some</td><td>4 out of 4</td><td>True and felicitous</td></tr>
<tr><td rowspan="3">Fillers</td><td>16</td><td rowspan="3">16 all and 16 any, balanced across truth values</td><td>only some</td><td>2 out of 4</td><td>True and felicitous</td></tr>
<tr><td>8</td><td>only some</td><td>4 out of 4</td><td>False and infelicitous</td></tr>
<tr><td>8</td><td>only some</td><td>0 out of 4</td><td>False and infelicitous</td></tr>
</table>
