# Supplementary material for: Context-Sensitivity and Individual Differences in the Derivation of Scalar Implicature
Source: Front Psychol. 2018 Sep 20;9:1720. doi: 10.3389/fpsyg.2018.01720 (PMC6158351; doi:10.3389/fpsyg.2018.01720)
Supplement: Supplementary file 2 [file Table_2.pdf]

Supplementary Material

Context-sensitivity and Individual Differences in the Derivation of Scalar Implicature

Xiao Yang*, Utako Minai, Robert Fiorentino

*** Correspondence:** Xiao Yang: xiaoyang@ku.edu

Supplementary Tables

Table 2: Summary statistics of individual difference measures, including the mean, standard deviation (SD), minimum, and maximum value. The values shown here are raw values before transformation.

	Mean	SD	Min	Max
Count Span	66.53	15.08	39.22	94.00
Dot-pattern expectancy *d*-prime	3.33	.68	0.83	3.88
Stroop accuracy	-3.39	4.57	-16.25	2.50
AQ total score	20.11	5.86	8.00	36.00
PPVT-4	112.98	9.14	88	132
Author & Magazine Recognition task	15.13	8.33	2.50	38.00
