# Supplementary material for: Context-Sensitivity and Individual Differences in the Derivation of Scalar Implicature
Source: Front Psychol. 2018 Sep 20;9:1720. doi: 10.3389/fpsyg.2018.01720 (PMC6158351; doi:10.3389/fpsyg.2018.01720)
Supplement: Supplementary file 3 [file Table_3.pdf]

Supplementary Material

Context-sensitivity and Individual Differences in the Derivation of Scalar Implicature

Xiao Yang*, Utako Minai, Robert Fiorentino

*** Correspondence:** Xiao Yang: xiaoyang@ku.edu

Supplementary Tables

Table 3: Pairwise correlations (2-tailed) of individual difference measures (Significance codes: ** $p < 0.01$; * $p < 0.05$)

	Count span	Dot-pattern expectancy *d*-prime	Stroop accuracy	AQ total score	PPVT-4
Dot-pattern expectancy *d*-prime	.346**				
Stroop accuracy	.224	.221			
AQ total score	-.093	.036	.022		
PPVT4	.146	-.019	-.126	.046	
Author & Magazine Recognition task	-.084	-.060	-.148	.004	.252*
