# Supplementary material for: Context-Sensitivity and Individual Differences in the Derivation of Scalar Implicature
Source: Front Psychol. 2018 Sep 20;9:1720. doi: 10.3389/fpsyg.2018.01720 (PMC6158351; doi:10.3389/fpsyg.2018.01720)
Supplement: Supplementary file 4 [file Table_4.pdf]

Supplementary Material

Context-sensitivity and Individual Differences in the Derivation of Scalar Implicature

Xiao Yang*, Utako Minai, Robert Fiorentino

*** Correspondence:** Xiao Yang: xiaoyang@ku.edu

Supplementary Tables

Table 4: Pairwise correlations (2-tailed) of the composite scores of individual difference measures. *p* values are included in the brackets.

	Cognitive Resources	Socio-pragmatic Abilities
Socio-pragmatic Abilities	-.075 (p = .555)	
Language Skills	-.008 (p = .949)	.033 (p = .797)
