# Supplementary material for: Context-Sensitivity and Individual Differences in the Derivation of Scalar Implicature
Source: Front Psychol. 2018 Sep 20;9:1720. doi: 10.3389/fpsyg.2018.01720 (PMC6158351; doi:10.3389/fpsyg.2018.01720)
Supplement: Supplementary file 5 [file Image_1.pdf]

Supplementary Material

Context-sensitivity and Individual Differences in the Derivation of Scalar Implicature

Xiao Yang*, Utako Minai, Robert Fiorentino

*** Correspondence:** Xiao Yang: xiaoyang@ku.edu

Supplementary Figure

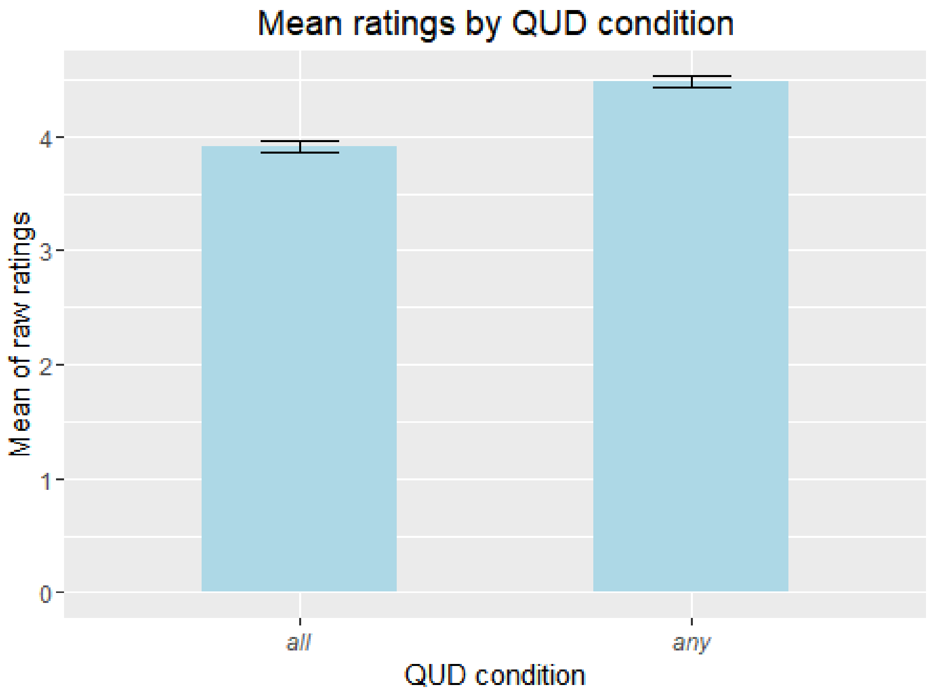

Supplementary Figure 1. Mean ratings in the *all* condition and in the *any* condition. Error bars illustrate Standard Error for each condition. For visualization purposes, the plot is based on raw ratings; the statistical analysis was performed on the odds ratio of increasing the ratings, as discussed in the Results of the manuscript.
